# Previous resistance exercise training mitigates progression of right ventricle dysfunction and remodeling in male rats with pulmonary arterial hypertension

**DOI:** 10.14814/phy2.70786

**Published:** 2026-02-23

**Authors:** Alexandre Martins Oliveira Portes, Anselmo Gomes de Moura, Franciany de Jesus Silva, Luiz Otávio Guimarães‐Ervilha, Maíra Oliveira de Freitas, Leôncio Lopes Soares, Bruna Aparecida Fonseca da Silva, Sebastião Felipe Ferreira Costa, Sara Caco dos Lúcio Generoso, Thiago de Souza Silva, Thainá Iasbik‐Lima, Luciano Bernardes Leite, Mariana Machado Neves, Emily Correna Carlo Reis, Antônio José Natali

**Affiliations:** ^1^ Department of Physical Education Federal University of Viçosa Viçosa Brazil; ^2^ Department of Biological Sciences Federal University of Ouro Preto Ouro Preto Brazil; ^3^ Department of General Biology Federal University of Viçosa Viçosa Minas Gerais Brazil; ^4^ Veterinary Department Federal University of Viçosa Viçosa Brazil

**Keywords:** AT/ET ratio, hypertrophy, myocyte contractile function, TAPSE

## Abstract

This study investigated whether previous resistance exercise training (RT) affects the progression of right ventricular dysfunction and remodeling in rats with severe pulmonary artery hypertension (PAH). Male Wistar rats were submitted to a RT protocol (i.e., ladder‐climbing) for 8 weeks, while controls remained in cages without exercising. Then, exercised rats were randomly divided into trained monocrotaline discontinued (TMD), and trained monocrotaline continued (TMC) groups. Subsequently, they received a single monocrotaline injection (i.e., 60 mg/kg) and the TMD group stopped RT, while the TMC group exercised for an additional 6‐week period. After euthanasia, right ventricle (RV) was dissected and processed for histological and single RV myocyte analyses. Previous RT increased physical effort tolerance, prevented pulmonary artery resistance augment (i.e., AT/ET) and mitigated the reduction in RV systolic function (i.e., TAPSE). RT also lessened impairments in single RV myocyte contractility and intracellular calcium transient (i.e., amplitude, and times to peak and relaxation) in the TMC group only. Moreover, RT inhibited adverse RV remodeling (i.e., hypertrophy and collagen deposition) in both trained groups. In conclusion, previous RT attenuates the progression of RV dysfunction and remodeling in rats with severe monocrotaline‐induced PAH, being the extension of protective effects dependent on the exercise training continuity.

## 1 INTRODUCTION

Pulmonary arterial hypertension (PAH) is a progressive disease exhibiting poor prognosis with a mean survival of around 2.8 years (Leber et al., 2021; Mocumbi et al., 2015; Montani et al., 2013). The initial insult involves pulmonary vasculature damage because of augmented oxidative stress and inflammation (Bowers et al., 2004; DeMarco, 2010; Freire et al., 2024; Xu et al., 2022). The consequent increases in pulmonary vascular resistance and pulmonary artery resistance generate direct overload to the right ventricle (RV), which causes its adverse remodeling expressed by hypertrophy, deposition of collagen and fibrotic tissue, dysfunction, and insufficiency (Colombo et al., 2013; Humbert et al., 2019; Maron et al., 2020; Mocumbi et al., 2015; Novelli et al., 2019; Okada et al., 2009; Shi et al., 2018; Takimoto & Kass, 2007; Talati & Hemnes, 2015).

Right ventricle dysfunction is secondary to deterioration of tissue complacence (i.e., increased stiffness) which impairs its capacity to contract and relax (Okada et al., 2009; Shi et al., 2018), and myocyte mechanical function that implement force and velocity to contraction and relaxation (Bers, 2002; Leite et al., 2025; Natali et al., 2015; Silva et al., 2021; Soares et al., 2019). Therefore, declines in myocyte contractility and intracellular Ca^2+^ availability and regulation are present in stable and severe PAH induced by monocrotaline (MCT) in rats (Leite et al., 2025; Natali et al., 2015; Silva et al., 2021; Soares et al., 2019, 2023). Moreover, changes in the intracellular Ca^2+^ transient kinetic and expression of ryanodine receptor type 2 (RyR2), sarcoplasmic reticulum Ca^2+^ ATPase type 2a (SERCA2a) and phospholamban (PLB) which regulates Ca^2+^ release from and sequestration into sarcoplasmic reticulum, respectively, are also reported in such model of severe PAH in rats (Leite et al., 2025; Moreira‐Gonçalves et al., 2015; Natali et al., 2015; Pacagnelli et al., 2016; Silva et al., 2021; Soares et al., 2019; Soares et al., 2023). RV dysfunction together with other central (i.e., pulmonary) and peripheral (i.e., skeletal muscle) damages results in low tolerance to physical exercise and functional capacity associated with inadequate quality of life and survival prognosis in PAH patients (Sahni et al., 2015; Zafrir, 2013).

Concerning treatments, regardless of progress, high mortality and morbidity persist, which requires combined therapeutic strategies for the reduction of pulmonary arterial pressure and RV overload (Humbert et al., 2022; Lajoie et al., 2017), since RV function is fundamental to survival in PAH patients (Chin et al., 2005; Haddad et al., 2008; La Gerche & Claessen, 2015). In this matter, different types of physical exercise have been shown to be safe and beneficial to patients with PAH (González‐Saiz et al., 2017; Grünig et al., 2019, 2021; Morris et al., 2023; Yan et al., 2021), because it helps maintain their cardiac functionality (Pandey et al., 2015; Yan et al., 2021), improving exercise capacity, quality of life, and reducing symptomatology (Ozemek et al., 2019). Considering that PAH is diagnosed mainly when patients are in advanced stages and RV impairment is already confirmed, it is of relevance to know if exercise preconditioning provides a RV environment that positively changes the ordinary disease's development.

In this way, few studies have tested such hypothesis in experimental severe PAH (Colombo et al., 2013; Nogueira‐Ferreira et al., 2016; Pacagnelli et al., 2016). They reported that previous aerobic exercise training provides cardiopulmonary functional and structural protection, thus delaying the evolution of MCT‐induced PAH. Nevertheless, whether previous resistance training (RT) elicits similar protective effects remains unknown. Therefore, observing that RT has proved beneficial to RV function and structure in experimental stable PAH (Soares et al., 2023), we investigated whether previous RT affects the progression of RV dysfunction and remodeling in male rats with severe MCT‐induced PAH.

## 2 METHODS

### 2.1 Study design

Male Wistar rats (Age: 8 weeks; body weight: ~250 g) were obtained from the animal house at the Federal University of Viçosa‐MG, Brazil. The animals were housed in transparent polypropylene cages (i.e., five animals per cage) in a room with controlled temperature (~22°C) under a 12/12 light/dark cycle and received filtered water and commercial food (Nuvilab CR‐1, Colombo, PR, Brazil, Cat. No. 100110007) ad libitum. Rats were randomly divided into four groups: sedentary control (SC; *n* = 14), sedentary monocrotaline (SM; *n* = 14), trained monocrotaline discontinued (TMD; *n* = 14), and trained monocrotaline continued (TMC; *n* = 14). The sedentary groups (SC and SM) remained in their cages without exercise training throughout the experimental period. The TMD and TMC groups performed an RT program for 8 weeks, and at the end of such a period received one injection of monocrotaline (see below). Following, the TMD group interrupted the exercise training while the TMC group performed another 6 weeks of RT.

### 2.2 Maximum carrying load test and resistance training protocols

The RT program was adapted from Hornberger and Farrar (Hornberger Jr. & Farrar, 2004). RT was performed on a vertical ladder climbing (i.e., 118 cm; 18 cm wide; 2 cm distance between the steps of the grid, and 80° of inclination). Initially, all rats underwent the climbing familiarization process during 2 weeks, three times a week, on non‐consecutive days, as follows: (1) on the first week, rats were stimulated to perform 8–12 climbs and remain at the top of the stairs in a polypropylene cage for a 2‐min resting; and (2) on the second week, the rats performed 3 climbs carrying a load container (~15 g) attached to the tail, but with no load added. Familiarization was confirmed by the end of the second week, when rats performed 3 consecutive climbs voluntarily.

The initial maximum carrying load test was performed 48 h after familiarization. The rats were weighed, and the first climb was performed carrying an additional load of 75% of body mass. Subsequently, the carrying load was progressively increased by an additional 30 g at each climbing, interspersed with a 2‐min resting interval. This procedure was repeated successively until the rat could not complete the climb. The load carried on the last complete climb was considered the maximum load and was used as a physical effort tolerance index. To update the training load, the maximum carrying load test was repeated in the 4th and 8th weeks of RT before MCT or saline injection, and weekly after 20 days of injections until euthanasia.

The RT sessions initiated 48 h after the maximum carrying load test and were performed three times/week, on non‐consecutive days, for a period of 8 weeks by the TMD group and 14 weeks by the TMC group. The RT protocol consisted of four to nine climbs carrying progressive loads. During the first four climbs, the rats carried 50%, 75%, 90%, and 100% of the maximum carrying load, respectively. Subsequently, 30 g was added progressively to each climb until completing nine climbs.

### 2.3 Doppler echocardiography

The first Doppler echocardiography was performed at the end of the 8th week of RT, before application of monocrotaline or saline, and the second and third assessments were performed 24 and 42 days after injections. In summary, the animals were anesthetized (3% isoflurane in 100% oxygen for induction and 1.5% for maintenance, in a constant flow of 1 L/min; Isoflurane, BioChimico, RJ, Brazil) and the images were obtained while the animals were in the lateral decubitus position. Two‐dimensional studies with a fast‐sampling rate of 120 frames per second in M mode were performed using the MyLabTM30 ultrasound system (Esaote, Genoa, Italy) and 10 MHz nominal frequency transducers.

The two‐dimensional transthoracic echocardiogram and M Mode were obtained at 200 mm/s sweep rate adjusted according to heart rate. The values of RV acceleration time (AT) and ejection time (ET) were obtained using Doppler pulsatile. The assessment of RV systolic function was measured using the tricuspid annular plane systolic excursion (TAPSE). For this, the M mode was used with the cursor positioned on the side of the annular plane of the tricuspid. The shortening from base to apex was measured during systole, identifying the lateral annulus of the tricuspid valve, the end diastolic and systolic distance. The collection of images was performed according to the recommendations of the American Society of Echocardiography (Sahn et al., 1978). The following parameters related to RV were evaluated: systolic function through TAPSE; AT and ET, and later, the AT/ET ratio was calculated.

### 2.4 Induction of pulmonary arterial hypertension

The induction of pulmonary arterial hypertension was performed by injecting intraperitoneally a dose of 60 mg/kg body weight of monocrotaline (Sigma‐Aldrich, St Louis, MO, USA) dissolved in saline (140 mM NaCl; pH 7.4) (Benoist et al., 2012). Controls rats (SC) received the same volume of saline solution (NaCl 140 mM; pH 7.4).

### 2.5 Sample collection

Rats were euthanized by decapitation using a guillotine specifically designed for rodents (Insight EB 271, Ribeirão Preto, SP, Brazil) after monocrotaline injection, when rats in the SM group showed characteristic signs of heart failure (e.g., weight loss, dyspnea, and piloerection; means of 43 days). We observe that two rats of the SM group died overnight during the experimental period and were not evaluated. The rats in the other groups were euthanized on days matching those in the SM group (i.e., one rat of each group euthanized on the same day). After euthanasia, the heart and RV were dissected, weighed and processed according to the analysis of interest (see below).

### 2.6 Isolation of right ventricular myocytes

Right ventricular myocytes were isolated as described previously (Natali et al., 2015). In brief, the heart was removed and placed in a retrograded perfusion system (Langendorff) through the ascending aortic and perfused with the constant flow of the Tyrode solution containing (in mM; Sigma‐Aldrich, St Louis, MO, USA): 130 NaCl, 1.43 MgCl_2_, 5.4 KCl, 0.75 CaCl_2_, 5.0 HEPES, 10.0 glucose, 20.0 taurine (Cat. No. 86239) and 10.0 creatine (Cat. No. C0780), pH 7.4 until the blood vessels were clear of blood (~6 min). The Tyrode solution was exchanged to Tyrode solution containing ethylene glycol tetraacetic acid (EGTA; 0.1 mM) for 6 min for the rupture of the intercalated discs and chelation of Ca^2+^.

After, the heart was perfused with Tyrode solution containing 1 mg/mL collagenase type II (Worthington, Lakewood, NJ, USA, Cat. No. LS004177, Species source: Clostridium histolyticum) and 0.1 mg/mL protease (Sigma‐Aldrich, St Louis, MO, USA, Cat. No. P6147) for about 10 min for digestion of extracellular collagen fibers. Then, the RV was dissected and placed in a flask containing enzymatic solution (collagenase and protease) and the cells were mechanically separated by shaking the flask for 5 min. The dispersed cells were separated from the non‐dispersed tissue by centrifugation and filtration. Following, the obtained myocytes were resuspended in Tyrode solution and were stored at 5°C until use. Isolated myocytes were used within 2–3 h after isolation. The solutions used in the isolation procedure were oxygenated (O_2_ 100%‐White Martins, Rio de Janeiro‐RJ, Brazil) and maintained at 37°C.

### 2.7 Measurement of myocyte contractile function and intracellular Ca^2+^ transient

The contractions of isolated myocytes were measured by using an edge detection system (Ionwizard, IonOptix, Milton, MA, USA) mounted on an inverted microscope (Nikon Eclipse‐TS100, Japan) as described by others (Natali et al., 2015). In summary, isolated myocytes were accommodated in an experimental chamber mounted on an inverted microscope and superfused with Tyrode's solution containing in mM (Sigma‐Aldrich, St Louis, MO, USA): 137 NaCl, 5.4 KCl, 0.33 NaH_2_PO_4_, 0.5 MgCl_2_, 5 HEPES, 5.6 glucose, and 1.8 CaCl_2_, pH 7.4 with 5 N NaOH, at 37°C. Subsequently, the myocytes were externally stimulated at frequencies of 1, 3, 5, and 7 Hz (40 V, 5 ms) using a pair of external electrodes placed inside the chamber (Myopacer, IonOptix, Milton, USA).

Individual myocytes were then visualized on a monitor using a CCD camera (Myocam, IonOptix, Milton, USA) and the images of cell contractions were recorded. The cell shortening and relaxation parameters of myocytes showing clear and regular striations without presenting spontaneous contractions were registered by the edge detection system and were analyzed using software (IonWizard 6.3; IonOptix, Milton, USA). Myocyte length and width were obtained from the video images.

The intracellular Ca^2+^ transient was determined as described elsewhere (Chang et al., 2019). Briefly, single myocytes were loaded with fura‐2 am (Thermo Fisher, Waltham, MA; 3Mm, USA, Cat. No. F1221) and placed in an experimental chamber, mounted on the same inverted microscope and bathed with the same Tyrode's solution used for cell contraction measurement. After, they were submitted to epifluorescence at 37°C in a dark room and were electrically stimulated (40 V, 5 ms) at 1, 3, 5, and 7 Hz frequencies (MyoPacer, IonOptix Milton, USA). The intracellular Ca^2+^ transient was measured using an excitation system dual for detection of fluorescence excited by UV light with the ratio of the emitted fluorescence at 510 nm in response to the excitation at 340 and 380 nm. The fluorescence recorded was the ratio between the 340 and 380 nm excitations (340 nm/380 nm ratio). Imaging was performed using a Nikon Eclipse TS100 microscope (Melville, NY, USA) equipped with a 40×/1.30 oil‐immersion S Fluor objective (Nikon, Melville, NY, USA). The intracellular Ca^2+^ transient parameters were analyzed using specific software (IonWizard 6.3; IonOptix, Milton, USA).

### 2.8 Histological processing

Right ventricle samples were immersed in Karnovsky's fixative solution (2.5% glutaraldehyde, 4% paraformaldehyde in 0.1 M phosphate buffer, pH 7.4) for 24 h (Karnovsky, 1965). After the fixation period, tissues were dehydrated in a crescent series of ethanol (70%, 80%, 90%, and 100%) and embedded in methacrylate (Historesin®, Leica Microsystems, Nussloch, Germany, Cat. No. 10695–0002). Semi‐serial sections of 3 μm thickness were obtained using a rotating microtome (RM 2255, Leica Biosystems, Nussloch, Germany), mounted on histological slides, and stained with Sirius Red (Sigma‐Aldrich, St Louis, MO, USA, Cat. No. 365548) to count collagen fibers. Digital images of slides stained with Sirius Red were obtained using a polarized light microscope (Olympus BX‐53, Tokyo, Japan) connected to a digital camera (Olympus DP73, Tokyo, Japan).

### 2.9 Morphometric analyses

To estimate the proportion of RV components, we used a grid with 266 intersections over 10 random images of each animal (20× magnification), totalizing 2.660 points per animal, using the Image J® software (National Institute of Health, USA). The percentage of points in each component was calculated using the formula: volumetric proportion (%) = (number of points in the interest structure/2.660 total points) × 100. The quantification of collagen was performed using a specific color identification tool using the Image‐pro Plus 4.5 software (Media Cybernetics, Silver Spring, MD, USA).

### 2.10 Statistical analysis

The data were submitted to the Shapiro–Wilk test to verify distribution. For parametric data, one‐way ANOVA was performed, followed by Tukey's post hoc test for multiple comparisons. For non‐parametric data, Kruskal–Wallis was used, followed by Dunn's post hoc test for multiple comparisons. Intragroup temporal assessments were performed using repeated measures ANOVA, followed by Tukey's post hoc, in parametric data. For non‐parametric data, the Friedman test, followed by Dunn's post hoc test, was used. Quantitative data are presented as means ± SD, while qualitative data are presented as percentages. An alpha error probability of up to 5% was considered. The statistical tests and the numbers of animals used in each parameter evaluated are displayed in tables and figures. All analyses were performed using the GraphPad Prism software version 8.0.2.

## 3 RESULTS

### 3.1 Physical effort tolerance

**FIGURE 1:**
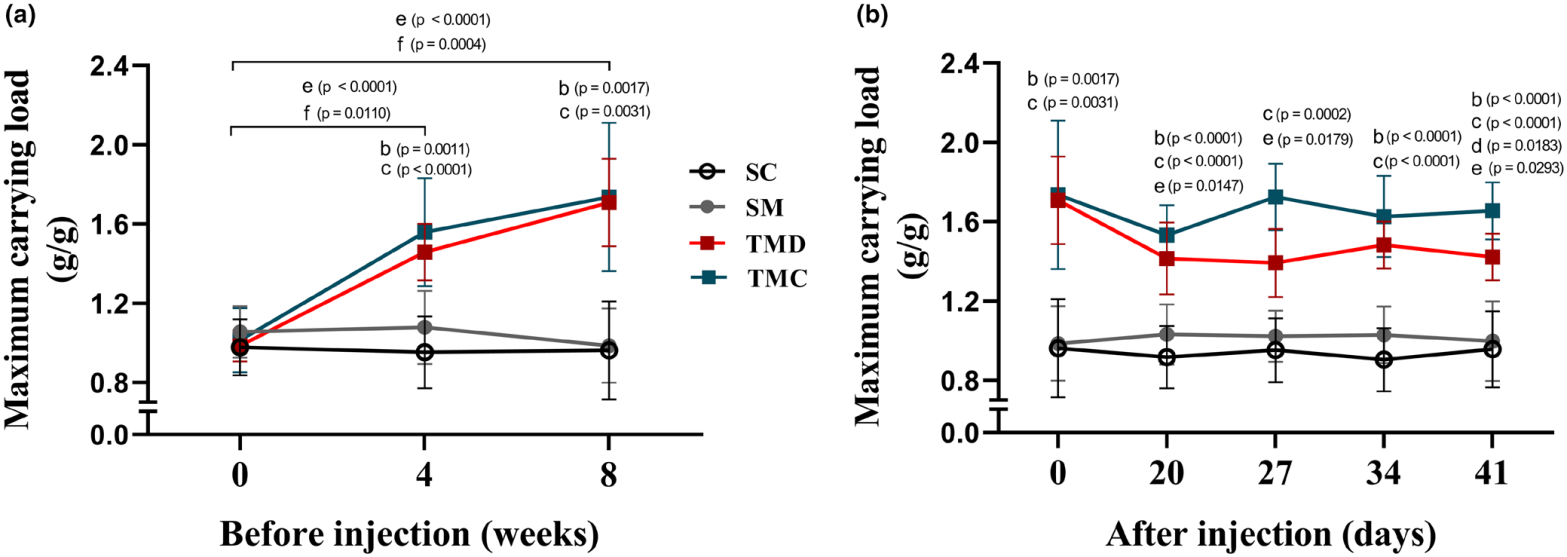
Physical effort tolerance. (a) Relative carrying load before injection. (b) Relative carrying load after injection. SC, sedentary control; SM, sedentary monocrotaline; TMC, trained monocrotaline continued; TMD, trained monocrotaline discontinued. Data are means ± SD of 12–14 rats in each group. One‐way repeated measures ANOVA followed by the Tukey post hoc test to compare with day or Week 0. One‐way ANOVA followed by the Tukey post hoc test (Week 4, Days 20, 34, and 41). Kruskal–Wallis followed by Dunn's post hoc test for the other quantitative analysis. ^b^TMD vs. SM, ^c^TMC vs. SM, ^d^TMC vs. TMD, ^e^TMD vs. day or week 0, ^f^TMC vs. week 0.

Before injections (i.e., monocrotaline or saline), at the beginning of the experiment (i.e., Week 0), there were no differences between groups regarding maximum carrying load (Figure 1a). However, in Weeks 4 and 8, trained groups (i.e., TMD and TMC) showed higher maximum loads compared with those of week 0 and of the SM group. After injections, the maximum load (Figure 1b) remained higher in trained groups than in the SM group. Nonetheless, the TMD group presented reductions on Days 20, 34, and 41 compared with Day 0 and was not different from the SM group on Day 27. Additionally, the TMC group exhibited higher values compared to TMD on Day 41 and maintained values compared with Day 0. There were no differences between SC and SM groups over the experimental period.

### 3.2 Whole animal and organ parameters

The presence of PAH is noted by changes in RV and cardiac proportions (Table 1). In fact, there were no differences between groups for the initial body weight, LV + interventricular septum weight and tibial length. The final body weight was lower in the SM group compared with the SC group. However, the SM group presented higher heart and RV weights as well as its ratio to tibial length and Fulton's index than the SC group. Previous RT, on the other hand, prevented those changes in heart and RV weights and ratios to tibial length in both TMD and TMC groups. The increase in Fulton's index was avoided in the TMC group only.

**TABLE 1:** Whole‐animal and organ morphological parameters.

Parameters	SC	SM	TMD	TMC
Initial body weight g	244.00 ± 23.40	240.92 ± 22.84	260.31 ± 20.45	242.46 ± 17.24
Final body weight, g	433.38 ± 57.68	375.85 ± 43.48*	376.00 ± 35.24	376.69 ± 29.67
Heart weight, g	1.31 ± 0.08	1.65 ± 0.05*	1.49 ± 0.11**	1.38 ± 0.10**
LV + interventricular septum, g	0.97 ± 0.07	0.93 ± 0.08	0.91 ± 0.06	0.86 ± 0.08
RV weight, g	0.17 ± 0.01	0.44 ± 0.03*	0.36 ± 0.06**	0.28 ± 0.03** ^,^ ***
Fulton's index, g/g	0.18 ± 0.02	0.47 ± 0.07*	0.40 ± 0.05	0.33 ± 0.04**
Tibial length, mm	41.48 ± 1.48	42.20 ± 1.37	42.23 ± 1.84	42.23 ± 0.64
Heart weight/tibial length, mg/mm	31.28 ± 2.45	39.20 ± 1.88*	35.36 ± 1.48**	32.67 ± 2.11**
RV/tibial length, mg/mm	4.12 ± 0.23	10.33 ± 0.67*	8.60 ± 1.07**	6.74 ± 0.80** ^,^ ***

*Note*: Data are means ± SD of 12–14 rats in each group. ANOVA one‐way followed by the Tukey post hoc test.

Abbreviations: LV, left ventricle; RV, right ventricle; SC, sedentary control; SM, sedentary monocrotaline; TMC, trained monocrotaline continued; TMD, trained monocrotaline discontinued.

* *p* < 0.05 vs. SC.

** *p* < 0.05 vs. SM.

*** *p* < 0.05 vs. TMD.

### 3.3 Pulmonary artery resistance and RV systolic function

**FIGURE 2:**
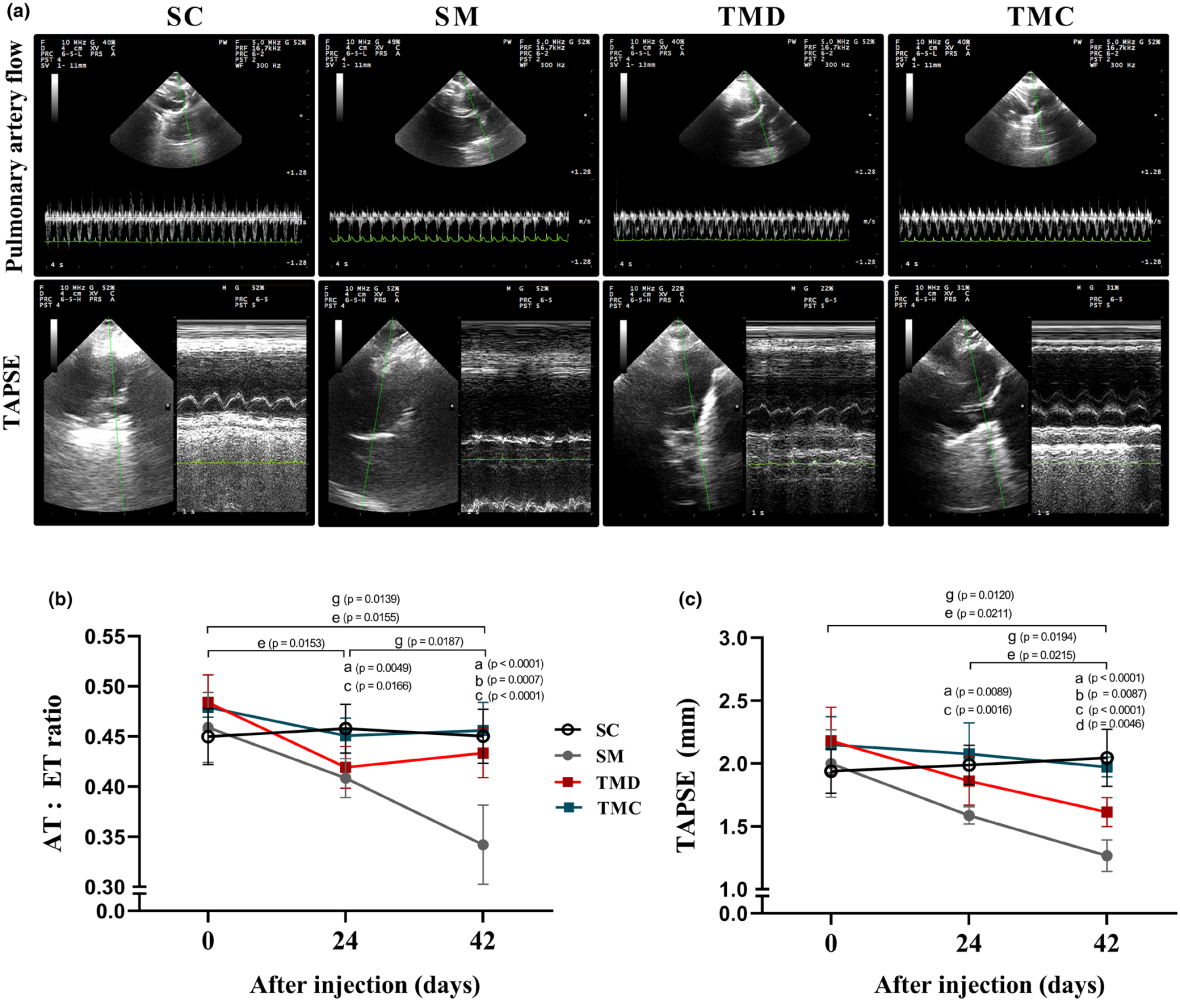
Pulmonary artery resistance and right ventricular systolic function. (a) Representative echocardiographic images of pulmonary artery flow (upper panel) and tricuspid annular plane systolic excursion (lower panel) registered 42 days after injections. (b) Ratio of flow acceleration time to ejection time (AT/ET). (c) Tricuspid annular plane systolic excursion (TAPSE). SC, sedentary control; SM, sedentary monocrotaline; TMC, trained monocrotaline continued; TMD, trained monocrotaline discontinued. Data are means ± SD of 12–14 rats in each group. One‐way repeated measures ANOVA followed by Tukey. One‐way ANOVA followed by Tukey post hoc test. ^a^SM vs. SC, ^b^TMD vs. SM, ^c^TMC vs. SM, ^d^TMC vs. TMD, ^e^TMD vs. day 0, ^g^SM vs. 0 or 24 days.

Pulmonary arterial hypertension was characterized by reductions in pulmonary artery flow (Figure 2a, upper panel) and TAPSE (Figure 2a, lower panel). Indeed, there were no differences between groups for the AT/ET ratio before monocrotaline or saline injection, after the previous RT (Figure 2b, Day 0). After injections, however, the SC group maintained the AT/ET ratio until Day 42, while the SM group showed lower values on Days 24 and 42 compared with Days 0 and 24, respectively, and with the SC group. The TMD group showed lower values on Days 24 and 42 than on Day 0; however, it showed maintenance between Days 24 and 42 and a higher AT/ET ratio compared with the SM group on Day 42. The TMC group maintained the AT/ET values throughout the experiment and showed higher values than the SM group on Days 24 and 42.

Concerning the TAPSE (Figure 2c), there were no differences between groups before injections, after the previous RT (Day 0). After injections, the SM group exhibited a tendency to reduce TAPSE on Day 24, compared with Day 0 (*p* = 0.054). However, on Day 42, the SM group presented lower TAPSE compared with Days 0 and 24, and lower values than those of the SC group on Days 24 and 42. The TMD group displayed lower TAPSE on Day 42 compared with Days 0 and 24 and compared with the TMC group. However, it had higher values than those of the SM group. The TMC group, nevertheless, maintained the TAPSE values throughout the experiment, and presented higher values than those of the SM group on Days 24 and 42 and compared with the TMD group on Day 42.

### 3.4 Single RV myocyte contractile function and intracellular Ca^2+^ transient

**FIGURE 3:**
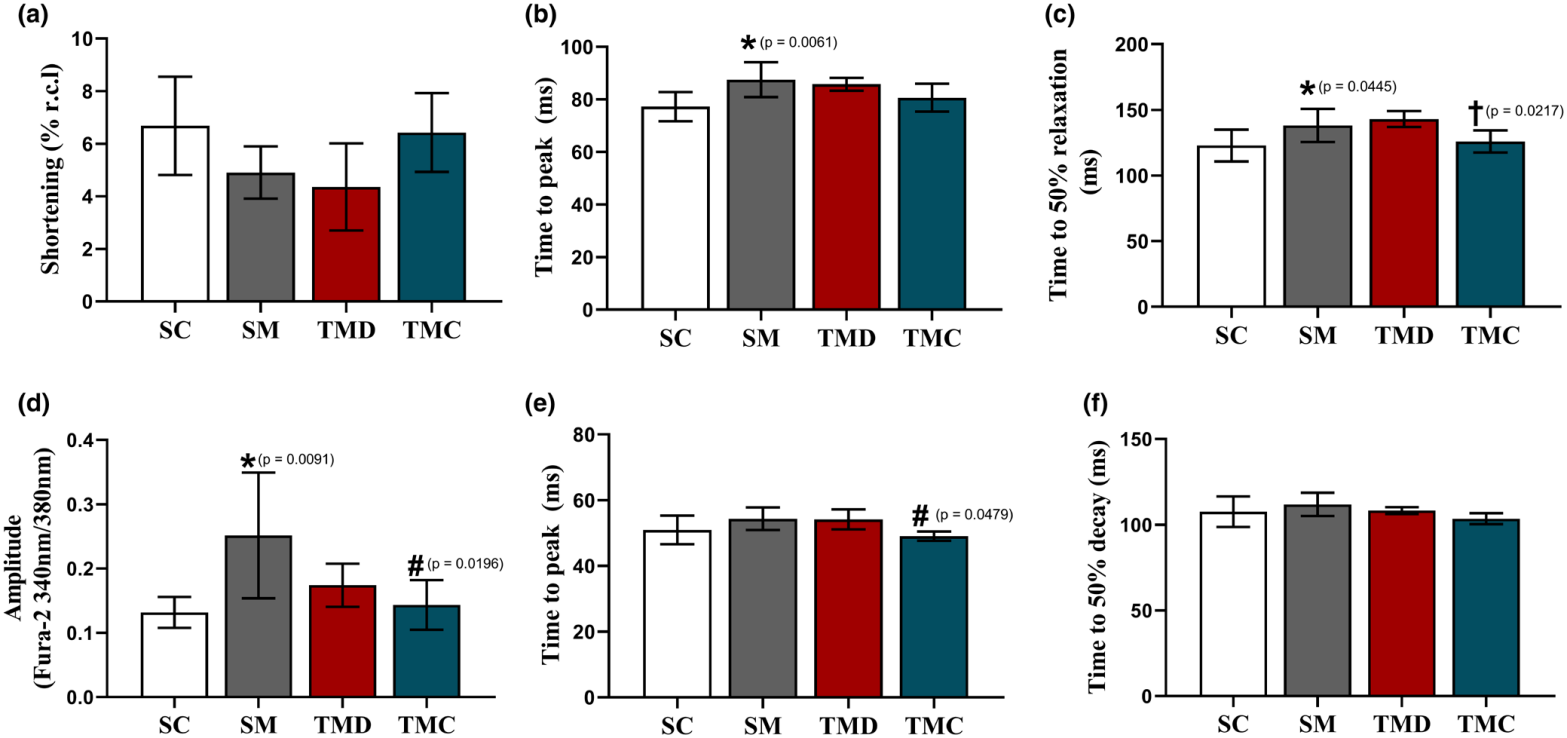
Right ventricular single myocyte contractile function and intracellular Ca^2+^ transient. (a) Amplitude of shortening. (b) Time to peak of shortening. (c) Time to 50% relaxation. (d) Amplitude of Ca^2+^ transient. (e) Time to peak of Ca^2+^ transient. (f) Time to 50% decay. SC, sedentary control; SM, sedentary monocrotaline; TMD, trained monocrotaline discontinued; TMC, trained monocrotaline continued. Resting cell length (r.c.l.). Data are means ± SD of 6–15 cells per animal from 6 to 7 rats in each group. One‐way ANOVA followed by the Tukey post hoc test for time to peak. Kruskal–Wallis followed by the Dunn's post hoc test for the other quantitative analysis. *vs. SC, ^#^vs. SM, ^†^vs. TMD.

The effect of the disease on the amplitude of shortening did not reach statistical significance (Figure 3a). However, the TMC group exhibited values like those of the SC group. In addition, the time to peak of shortening (Figure 3b) was higher in the SM group than in the SC group. Nevertheless, in both TMD and TMC groups such increase was not found. Moreover, the time to 50% relaxation (Figure 3c) was greater in the SM group compared with the SC group. Nonetheless, previous RT prevented such increase in the TMC, while in the TMD group the protection was not observed.

The amplitude of the intracellular Ca^2+^ transient was higher in the SM group compared with the SC group (Figure 3d). Previous TR, however, prevented such increases in the TMC group, though in the TMD group the protection was not present. In addition, time to peak of the intracellular Ca^2+^ transient (Figure 3e) was not affected by the disease. Nevertheless, previous RT reduced such time in the TMC group compared with the SM group. Furthermore, the time to 50% decay (Figure 3f) was not different between groups.

### 3.5 RV adverse remodeling

**FIGURE 4:**
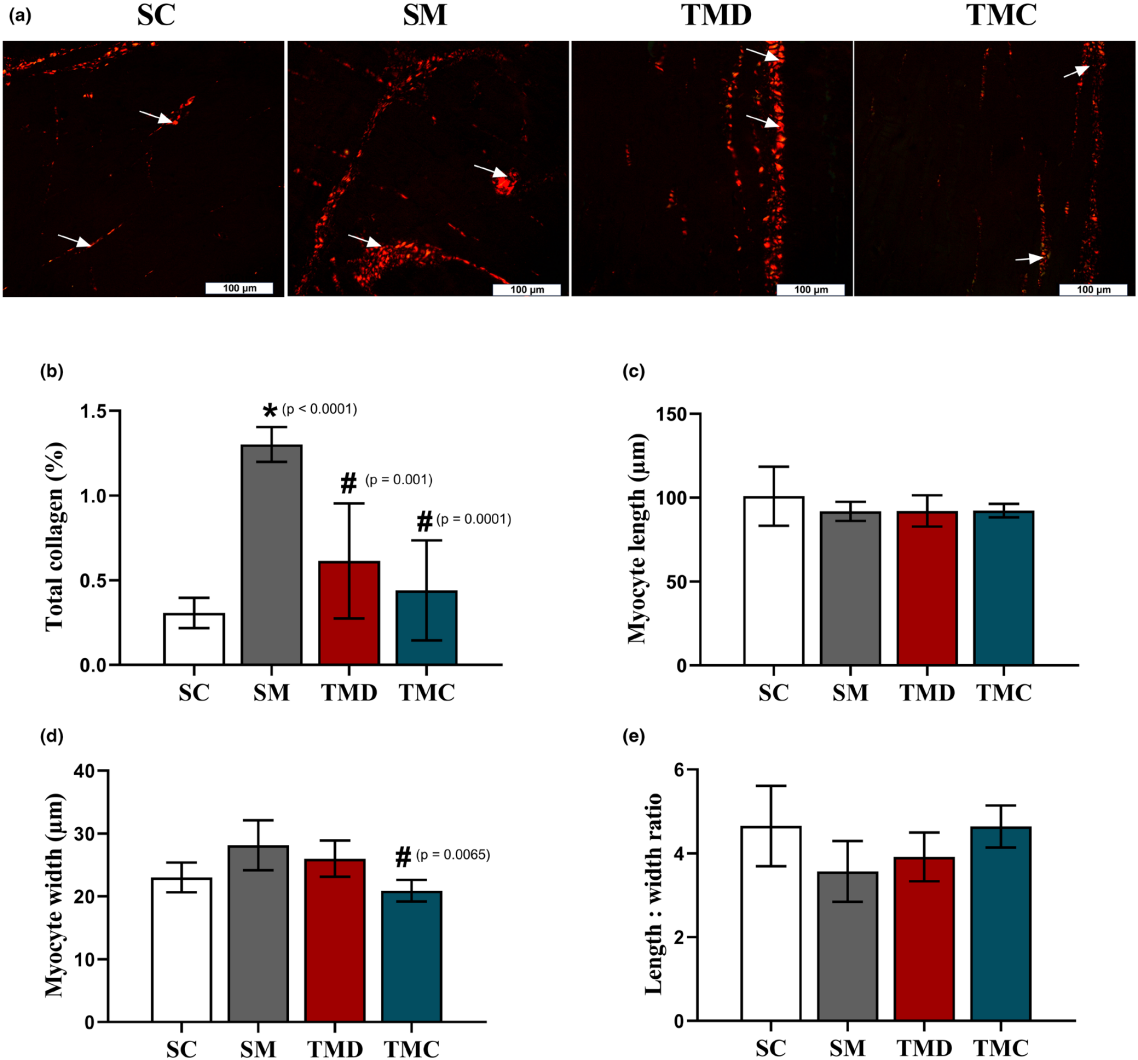
Right ventricular adverse remodeling. (a) Representative photomicrographs of RV tissue stained with Sirius red (white arrow: Collagen). (b) Percentage of total collagen. (c) Single myocyte length. (d) Single myocyte width. (e) Single myocyte length to width ratio. SC, sedentary control; SM, sedentary monocrotaline; TMD, trained monocrotaline discontinued; TMC, trained monocrotaline continued. Data are means ± SD of 10 images per animal for total collagen, and 10 cells per animal for cell length and width from 6 to 7 rats in each group. One‐way ANOVA followed by the Tukey post hoc test (total collagen). Kruskal–Wallis followed by Dunn's post hoc test (myocyte dimensions). *vs. SC, ^#^vs. SM, ^†^vs. TMD.

The monocrotaline injection caused RV remodeling (Figure 4a) by augmenting collagen deposition. Rats in the SM group exhibited a higher percentage of total collagen (Figure 4b), compared with the SC group. Previous RT, nonetheless, avoided the increase in total collagen in both TMD and TMC groups. Regarding cell dimensions, no difference between groups in myocyte length (Figure 4c) was found. The cell width was not affected by the disease (Figure 4d). Nevertheless, previous RT reduced such dimension in the TMC group, compared with the SM group. The effect of the disease on the length to width ratio did not reach statistical significance (Figure 4e), However, the TMC group exhibited values like those of the SC group.

## 4 DISCUSSION

Utilizing experimental severe PAH, we tested whether previous RT affects the progression of right ventricular dysfunction and adverse remodeling. Our results indicate that the protocol of physical exercise employed previously the disease development weakened damages to RV functional and structural remodeling. Specifically, it sustained right ventricular function at both organ and cellular levels mainly by mitigating impairments in myocyte contractility and intracellular calcium cycling as well as myocyte hypertrophy and collagen deposition, with more consistent effects found in hypertensive rats who continued exercising. These protective adaptations helped the conservation of exercise tolerance in hypertensive rats.

Initially, it is noteworthy that the injected monocrotaline greatly augmented pulmonary artery resistance (i.e., diminished AT/ET ratio) and hence the RV systolic function (i.e., TAPSE) was also notably shortened in sedentary hypertensive rats and such changes are characteristic in PAH installation (Leite et al., 2024; Soares et al., 2019; Tello et al., 2018). In this sense, these organ impairments were followed by inferior RV single myocyte contractile function, since slower velocities of shortening and relaxation were found in these rats. These functional alterations were aligned with RV morphological changes. For instance, hypertensive untreated rats exhibited hypertrophy and expanded Fulton index. Moreover, total collagen was elevated in these rats. These adaptations are linked to pathological remodeling because of increased oxidative stress and inflammation (Farahmand et al., 2004; Redout et al., 2010) that leads to PAH advancement (Aggarwal et al., 2013; Giordano, 2005; Leite et al., 2024; Peng et al., 2017; Takimoto & Kass, 2007; Türck et al., 2020; Zima & Blatter, 2006).

More relevantly, the RT employed previously to PAH induction improved the physical effort tolerance in hypertensive rats which was held during disease development being superior when animals continued exercising. Such beneficial adaptation might be associated not only with the conservation of central parameters (i.e., right heart structure and function) but also with peripheral adaptations to either resistance or combined exercise training, like sustenance of skeletal muscle structure and function and improved respiratory efficiency (Soares et al., 2023).

Respecting the beneficial adaptations to RV functionality caused by previous RT (i.e., TA/TE and TAPSE), it is reasonable that the mitigation of adverse remodeling by RT aided the maintenance of RV function. Indeed, the employed RT prevented augments in RV weight and myocyte width as well as in total collagen. It is known that the deposition of fibrotic tissue in the myocardium increases its stiffness, thus impairing its ability to contract and relax (Okada et al., 2009; Shi et al., 2018) which directly affects TAPSE. It is also probable that exercise training has guaranteed endothelial health (e.g., bioavailability of nitric oxide‐NO) and pulmonary artery morphology in trained hypertensive rats, because RT has been shown to increase eNOS activity (Ashor et al., 2015). Furthermore, in this model of PAH, RT has prevented the reduction in cardiopulmonary NO levels (Leite et al., 2025; Soares et al., 2023). Such vasodilatory effect alleviates mechanical pressure stress (Ahmed et al., 2014a; Alencar et al., 2014; Pei et al., 2011) in the pulmonary artery and hence its remodeling process (i.e., increases in wall thickness and reduction in lumen radius) (Ahmed et al., 2014b; Nassar et al., 2018; Zambelli et al., 2011) that straightly relieves the pressure overload imposed to the RV (Leong & Hikasa, 2019; Wang et al., 2013; Zhang et al., 2020). Our previous RT protocol avoided increases in pulmonary artery resistance (i.e., reduction in TA/TE) which favors RV performance and delays disease progression.

The preservation of RV activity was also supported by the effects of previous RT on myocyte contractile function and calcium cycling. For example, single RV myocytes from trained hypertensive rats who continued exercising had times to peak and relaxation equivalent to those in the control non‐hypertensive group while in sedentary hypertensive rats these parameters were significantly deficient. It is possible that RT has avoided impairments in the expression of channel proteins that regulate the sarcoplasmic reticulum Ca^2+^ release (i.e., RyR2) and sequestration (i.e., SERCA2a, pPLB), which is present in MCT‐induced PAH (Alencar et al., 2014; Moreira‐Gonçalves et al., 2015; Pacagnelli et al., 2016; Soares et al., 2023), though it was not assessed in this study. Nevertheless, our research group reported recently that RT maintains the RV levels of RyR2, pPLB^Ser16^ and SERCA2a in this model (Soares et al., 2023). Our data on the intracellular Ca^2+^ transient show that the amplitude of cell shortening did not change in sedentary hypertensive rats whereas the amplitude of the intracellular Ca^2+^ transient increased in these animals. This is a characteristic example of decreases in myofilament sensitivity to Ca^2+^ caused by the disease (Pacagnelli et al., 2016; Rain et al., 2013; Soares et al., 2023). In contrast, our previous RT mitigated such decline in myofilament sensitivity since in trained hypertensive rats (i.e., TMC group) the amplitude of the intracellular Ca^2+^ transient was like those in control non hypertensive rats.

It is important to observe that the preventive effects of previous RT on RV were more extensive in rats who continued exercising during the disease evolution. To illustrate, rats who discontinued the exercise protocol after MCT injection (i.e., TMD group) did not manage to conserve myocyte width and contractile function, although they benefited from protective effects of RT on pulmonary artery resistance, RV function and remodeling, and physical effort tolerance. Such results indicate that interruption of the exercise training results in losing gradually the benefits obtained (i.e., detraining).

Finally, this study has limitations, and attention is needed for interpreting its findings. First, the exercise intensity and workload are difficult to determine in as much as the animals' climbing speed is not controlled. Second, the treatment's duration was restrained by the MCT‐induced PAH time course, which may limit the generalizability of long‐term adaptations. Third, as the obliterative lesions seen in human PAH do not exist in the monocrotaline model (Gomez‐Arroyo et al., 2012), its clinical relevance is limited. And fourth, because only male rats were used, the applicability of our findings is limited and future studies on sex‐specific responses to the employed treatment in severe PAH are needed.

### 4.1 Conclusions

In conclusion, previous RT attenuates the progression of RV dysfunction and remodeling in rats with severe MCT‐induced PAH, being the extent of protective effects dependent on the exercise training continuity. These findings reinforce the relevance of regular RT, especially in the familial form of PAH since such action may help hinder the disease's severity or soon beginning.

## DISCLAIMERS

The supporters were not involved in the study design; data collection, analysis, and interpretation; the writing of the manuscript; and the decision to submit the manuscript for publication.

## AUTHOR CONTRIBUTIONS

**Alexandre Martins Oliveira Portes** and **Antônio José Natali**: Conceptualization, formal analysis, methodology, project administration, resources, writing original draft. **Anselmo Gomes de Moura**, **Franciany de Jesus Silva**, L**uiz Otávio Guimarães‐Ervilha**, **Maíra Oliveira de Freitas**, **Leôncio Lopes Soares**, **Bruna Aparecida Fonseca da Silva**, **Sebastião Felipe Ferreira Costa**, **Sara Caco dos Lúcio Generoso**, **Thiago de Souza Silva**, **Thainá Iasbik‐Lima**, **Luciano Bernardes Leite**, **Mariana Machado Neves**, and **Emily Correna Carlo Reis**: Methodology, data collection and analyses. **Antônio José Natali**, **Mariana Machado Neves**, and **Emily Correna Carlo Reis**: Writing review and editing, funding acquisition. All authors have read and agreed to the published version of the manuscript.

## FUNDING INFORMATION

This work was supported by the Fundação de Amparo à Pesquisa do Estado de Minas Gerais—Brasil (FAPEMIG) ‐ Grant No. APQ‐01485‐22, Conselho Nacional de Desenvolvimento Científico e Tecnológico—Brasil (CNPq) ‐ Grant No. 306956/2021–7, and Coordenação de Aperfeiçoamento de Pessoal de Nível Superior Brasil (CAPES)–Finance Code 001, and National Institute of Science and Technology ‐ INCT Physical (In)activity and Health, CNPq, Brazil ‐ Grant number 408899/2024–7.

## CONFLICT OF INTEREST STATEMENT

The authors declare no conflicts of interest.

## ETHICS STATEMENT

The experiments were conducted under protocols reviewed and approved by the Ethics Committee on Animal Use of the Federal University of Viçosa (CEUA‐UFV; Protocol No. 95/2018).

## Data Availability

The data discussed in this publication are available upon request from the corresponding author.
